# Intensification of hot Eurasian summers by climate change and land–atmosphere interactions

**DOI:** 10.1038/s41598-019-47291-5

**Published:** 2019-07-26

**Authors:** Tomonori Sato, Tetsu Nakamura

**Affiliations:** Hokkaido University, Sapporo, 060-0810 Japan

**Keywords:** Hydrology, Cryospheric science, Attribution, Atmospheric dynamics

## Abstract

Persistent abnormal hot weather can cause considerable damage to human society and natural environments. In northern Eurasia, the recent change in summer surface air temperature exhibits a heterogeneous pattern with accelerated warming around the Eastern European Plain and Central Siberia, forming a wave train-like structure. However, the key factors that determine the magnitude and spatial distribution of this summer temperature trend remain unclear. Here, a huge ensemble of general circulation model (GCM) simulations show that the recent summer temperature trend has been intensified by two factors: steady warming induced by external forcing and inhomogeneous warming induced by internal atmosphere–land interactions that amplify quasi-stationary waves. The latter is sensitive to both snow cover and soil moisture anomalies in the spring, suggesting the potential of land surface monitoring for better seasonal prediction of summer temperatures. Dramatic changes in the circumpolar environment, characterised by Eurasian snow variation and Arctic Ocean warming, collectively affect summertime climate via memory effects of the land surface.

## Introduction

**Figure 1:**
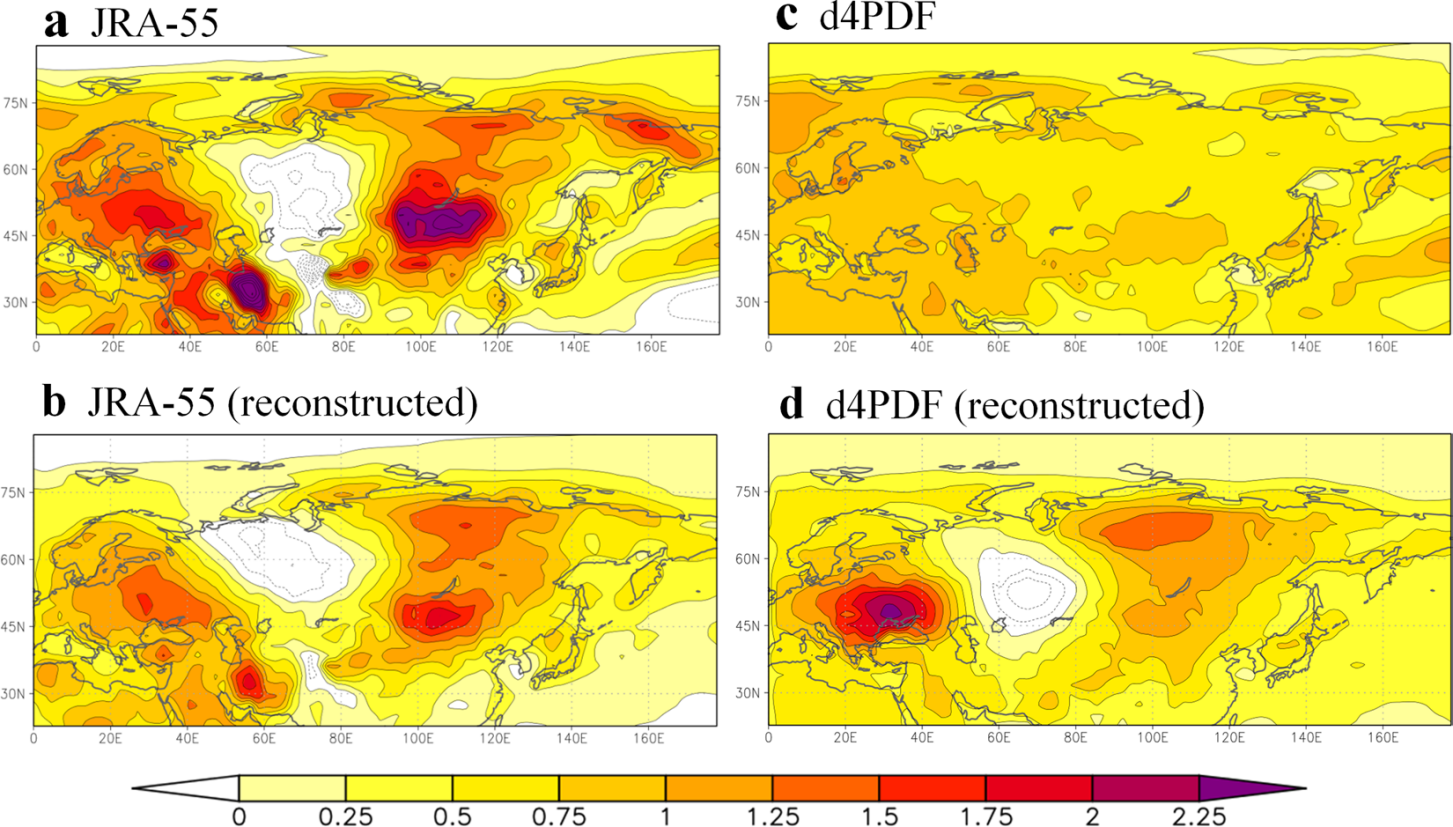
Summer SAT changes between the 2000s and the 1980s. JJA-averaged SAT change in the (**a**) JRA-55 and (**c**) d4PDF datasets (100 member average). (**b**,**d**) Reconstructed SAT differences between the 2000s and the 1980s by a linear combination of the first through third PCs derived from (**b**) JRA-55 and (**d**) d4PDF (see Methods). The 1980s and 2000s refer to 1981–1990 and 2001–2010, respectively.

Social awareness of abnormal weather is growing, particularly regarding the adverse effects of heat waves and droughts. Despite substantial efforts intended to mitigate climate change, the potential for the occurrence of extreme hot events is projected to increase in the future^1,2^. Over northern Eurasia, the difference in summer surface air temperature (SAT) between the 2000s and the 1980s shows a distinct spatial contrast, forming a continental-wide tripole pattern (Fig. 1a). Two regions with prominent SAT increases are around the Eastern European Plain and Central Siberia–Mongolia, in contrast to weak SAT changes around the West Siberian Plain. This tripole pattern has been recognised in many studies both in terms of summer mean SAT^3,4^ and hot extremes^5^ such as the Russian heat wave in 2010^6^ and multi-year heat waves in the 2000s around Mongolia^4,7^. In western Russia and Central Siberia–Mongolia, extreme hot events occurred with higher frequency than in the surrounding areas^5^. However, questions remain regarding the drivers of both the recent change in summer SAT and the occurrence of extreme hot events on a regional scale. Here, we examine the leading factors that alter summer SAT over northern Eurasia. We analyse a dataset derived from a 100-member ensemble experiment called the “database for Policy Decision making for Future climate change”^8^ (d4PDF, Methods), which comprises a 60-year integration (1951–2011) using MRI-AGCM3.2 (Meteorological Research Institute Atmospheric General Circulation Model version 3.2) driven by observed sea surface temperature (SST), sea ice, and natural and anthropogenic forcing.

## Results and Discussion

**Figure 2:**
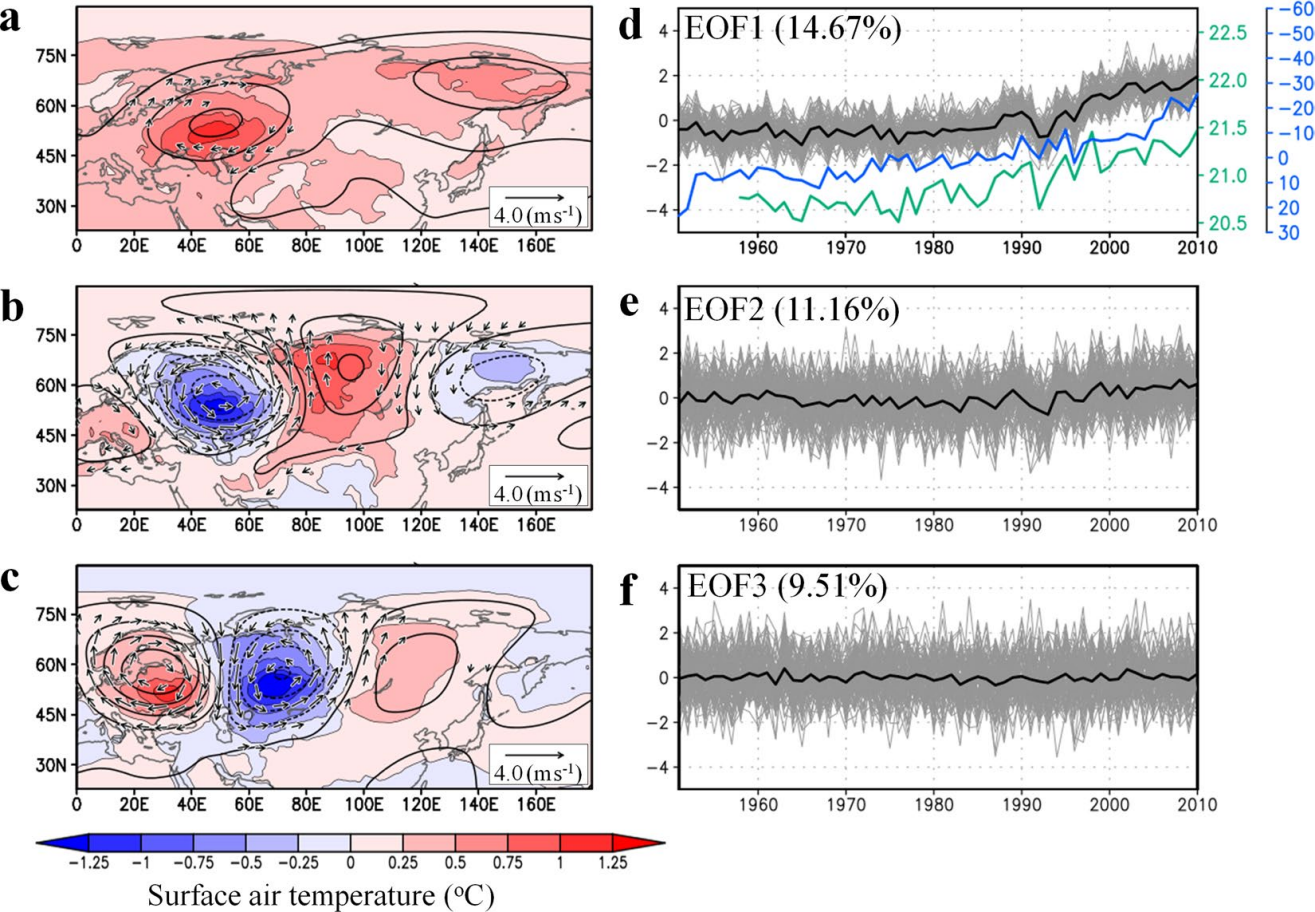
Three leading modes governing simulated summer SAT anomalies in d4PDF and their time series. (**a**–**c**) Regression patterns of JJA-averaged SAT (shading), 500 hPa geopotential height (contours: 5 m intervals), and wind vectors (m s^−1^) against the normalised score from each ensemble member (grey lines in **d**–**f**) for (**a**) first, (**b**) second, and (**c**) third PCs. Wind speeds <0.5 m s^−1^ are omitted. (**d**–**f**) Interannual variations of the normalised scores (grey) and their ensemble averages (black). Blue and green lines in (**d**) represent JJA means of sea ice area in the Arctic Ocean and northern hemispheric SAT, respectively. The sea ice area is presented as a percentage change relative to the 1951–2010 average.

**Figure 3:**
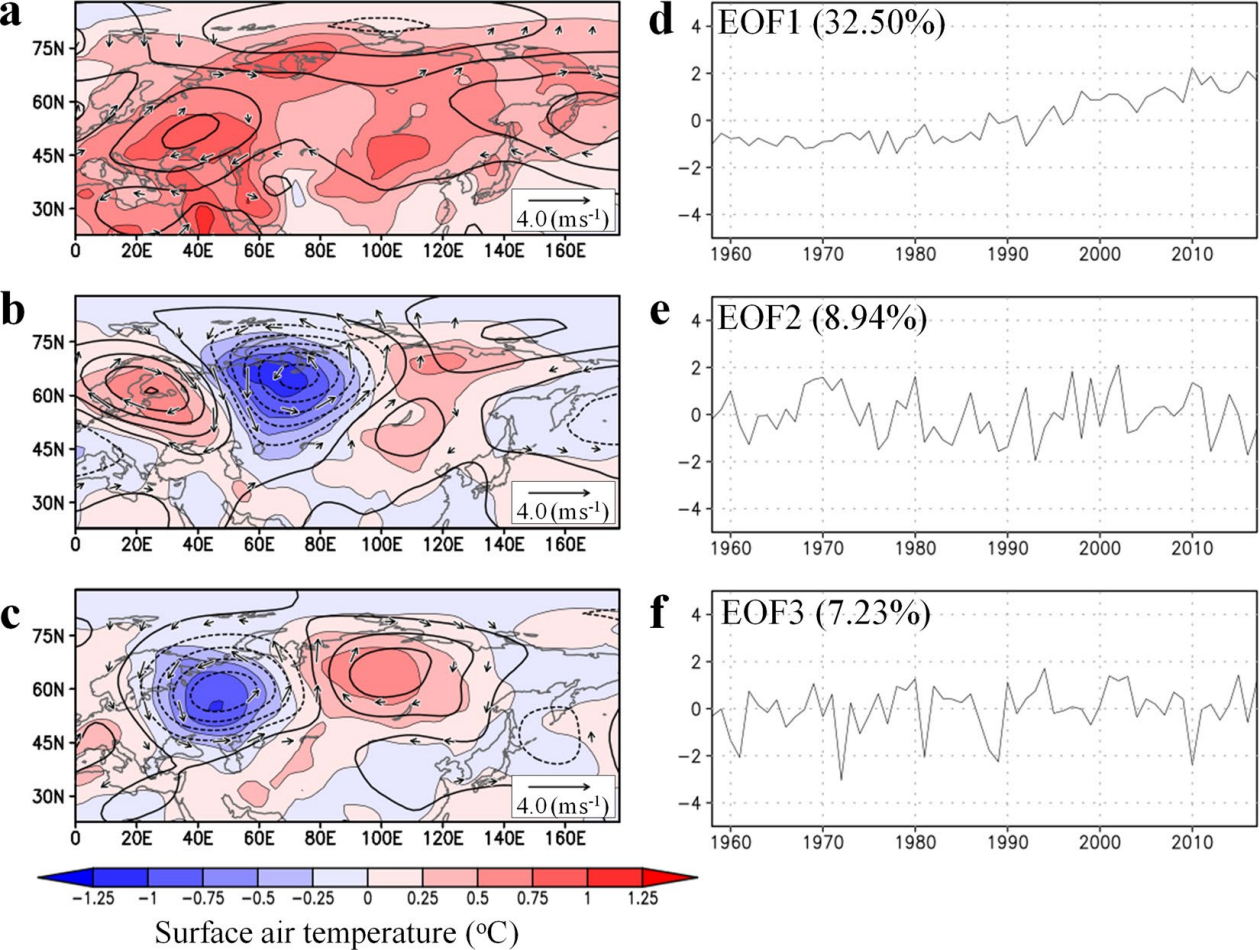
Three leading modes governing summer SAT anomalies in the JRA-55 reanalysis and their time series. The panels are the same as in Fig. 2 but for the EOF using the JRA-55 dataset. The period of analysis is 1958–2017.

The three leading modes for the June–July–August (JJA)-averaged SAT over mid-to-high latitudes of Eurasia were computed by empirical orthogonal function (EOF) analysis applied to d4PDF data (Fig. 2; Methods). Here, we use 6,000 snapshots of SAT derived from 100-member and 60-year-long data in the EOF analysis. The spatial pattern of the first principal component (PC1), which accounts for 14.67% of the total SAT variance, represents the continent-wide SAT increase with enhanced warming over western and northeastern Eurasia. The time series of the PC1 score shows accelerated warming after the early 1990s which is exhibited by all members. The interannual variation of the mean PC1 score is correlated strongly with northern hemispheric SAT (r = 0.91) and Arctic sea ice coverage (r = −0.80). Therefore, PC1 represents the response of Eurasian summer SAT to global climate change induced by external forcing and the resultant Arctic change, including SST increase and sea ice loss^9^. The PC1 is related to accelerated northward retreat of snow cover^10^ and increased water shortages in summer (Supplementary Fig. 1). The second and third principal components (PC2 and PC3), which together account for 20.6% of the total SAT variance, show longitudinal striped patterns over northern Eurasia, similar to the SAT trend in the reanalysis (Fig. 1a). The associated 500 hPa geopotential height fields indicate quasi-stationary wave propagation. The wave centres for PC2 and PC3 are shifted one-quarter phase. In contrast to PC1, interannual variations of the PC2 and PC3 scores appear to fluctuate irrespective of years, although weak increases in the PC2 score is recognised after the early 1990s. The random features of PC2 and PC3 indicate that the phase of wave propagation is determined independently of the prescribed SST variation; hence, the striped SAT pattern is likely attributable to internal dynamical processes of the coupled land–atmosphere system. The wave train patterns are also dominant when EOF analysis is applied to the detrended SAT by using JJA SAT deviation from the ensemble average (Supplementary Fig. 2). Thus, mid-latitude wave propagation plays a central role in the internal variability of summer SAT. These results are in accordance with earlier analyses based on atmospheric GCM (AGCM) experiments that suggested that in middle latitudes the effects of the coupled land–atmosphere system on Eurasian SAT variability are as strong as the effects of SST^11^. The leading patterns of the simulated SAT in d4PDF are similar to those in the reanalysis (Fig. 3) with warming trends in PC1 and wave-train patterns in PC2 and PC3. The huge ensemble output is advantageous in separating the effects of external forcing and internal variability^12^. The comparison between Figs 2 and 3 confirms that the recent warming in Eurasian summers is result of external forcing, while the internal forcing is responsible for the heterogeneous warming.

Figure 2 illustrates the successful decomposition of the recent SAT change into two components: one representing the effects of external forcing that robustly increase SAT irrespective of ensemble members, and the other representing the effects of internal dynamics that appear to fluctuate randomly on an interannual timescale. Figure 1b shows reconstructed SAT differences between the 2000s and 1980s computed from linear combinations of the first through third PC modes (Methods). The reconstructed SAT pattern resembles the reanalysis-based SAT change in that there are two areas of accelerated warming over the Eastern European Plain and Central Siberia and a region of negative SAT change over the Western Siberian Plain. This is a reasonable result because we used the optimised weighting coefficients for the linear combinations of the dominant PC modes. It is evident that the SAT increase around Mongolia has been underestimated, which is likely related to the absence of strong local feedback processes between soil moisture and the atmosphere^4^ in these leading modes. Figure 1 shows that wave train patterns, which fluctuate randomly, are essential for explaining the recent trend of summer SAT. Furthermore, the occurrence of recent extreme weather events in mid-latitude regions can be attributed to quasi-stationary wave propagation^13,14^.

**Figure 4:**
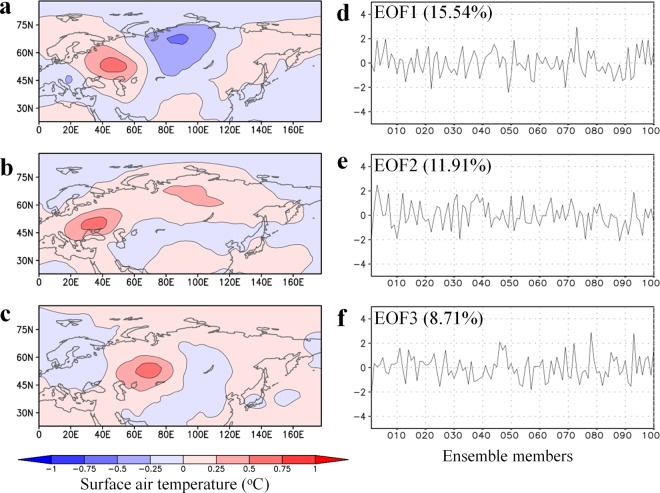
Leading patterns of summer SAT change between the 2000s and the 1980s in d4PDF. (**a**–**c**) Regression patterns of SAT change between the 2000s and the 1980s against normalised scores for (**a**) first, (**b**) second, and (**c**) third PCs. (**d**–**f**) Variations in normalised scores among ensemble members. The 1980s and 2000s refer to 1981–1990 and 2001–2010, respectively.

How are the SAT variations on interannual and decadal timescales related? Figure 4 displays the leading patterns of SAT differences on decadal timescales. Here, we conducted an EOF analysis for the SAT difference between 2000s and 1980s using 100 realisations derived from d4PDF. The wave train pattern appears to be the dominant mode of the summer SAT change on decadal timescales, which indicates decadal SAT and interannual SAT variations over northern Eurasia are closely related to each other. This generally agrees with earlier studies that pointed out the role of internal variability in multi-decadal trends^15,16^. Figure 4 shows that the PC scores of decadal SAT change are remarkably different among the members. This suggests that although the wave train pattern of SAT is a dominant mode on the decadal timescale, its sign is very diverse among the ensemble members. Because ensemble members in d4PDF are all constrained by observed SST and sea ice, decadal variations of SST and sea ice are not the sufficient drivers for the decadal SAT change. Therefore, the inter-member diversity of SAT variations on decadal timescales are also likely attributable to atmosphere and land processes.

**Figure 5:**
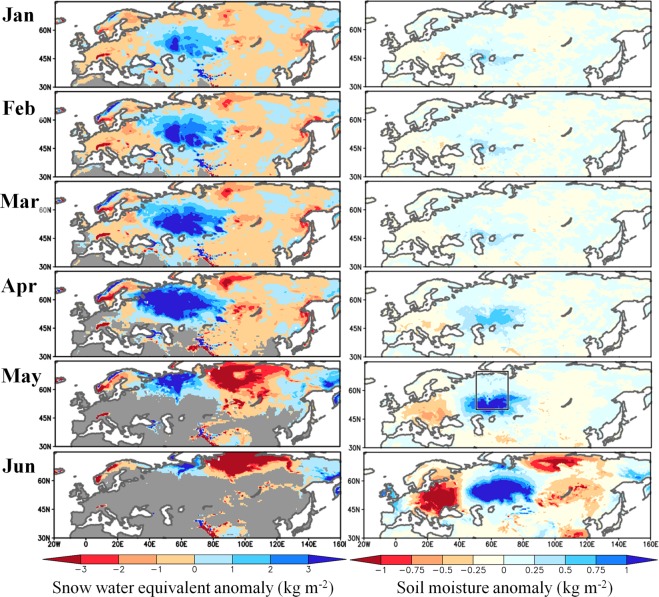
Seasonal variations of snow water equivalent and soil moisture associated with internal forcing. Monthly evolution of snow water equivalent (Left) and surface soil moisture (Right) anomalies reconstructed based on a linear combination of regression patterns for PC2 and PC3 (Methods) derived from d4PDF. The grey shading represents the area where climatological snow cover fraction is <0.1. Soil moisture in 0–10 cm depth is presented. The box indicates the area (50°–70°N, 50°–70°E) for statistical analysis in Fig. 6. The anomaly indicates the deviation from the 6,000 year average (60 years by 100 members).

To understand the processes underlying the recent SAT change, key factors that determine a phase of the wave train pattern are investigated. While many previous studies have emphasised the role of North Atlantic SST in initiating trans-Eurasian teleconnection patterns^17,18^, few have considered land surface processes^19^. It is widely known that soil moisture strongly regulates ground-to-atmosphere sensible heat flux^20^; therefore, positive feedback between the land and the atmosphere can amplify extreme heat and drought events^18,21–23^. Land surface anomalies evolve more slowly than atmospheric anomalies, which means the impact of surface anomalies on the atmosphere might appear after a time lag^21,22,24–27^. Figure 5 shows combined regression patterns of surface anomalies with respect to the PC2 and PC3 scores (Methods), which represent surface anomalies associated with internal forcing. During the preceding winter, high values of snow water equivalent extend around the Western Siberian Plain, which indicate large amounts of water are supplied to the soil layers during the spring snowmelt. Wetter soil starts to emerge in May when the snow melt is finished in the mid-latitude areas. A low 500 hPa geopotential height anomaly develops in May over the Western Siberian Plain where the underlying soil is wet (Supplementary Fig. 3). Positive feedback between soil moisture and the atmosphere further intensifies the positive soil moisture anomaly in combination with the intensified mid-tropospheric low pressure anomaly. The wave train pattern, which resembles the decadal SAT change, develops in June. These results provide evidence supporting earlier studies that have claimed the Arctic winter climate influences mid-latitude summer climate^28,29^.

**Figure 6:**
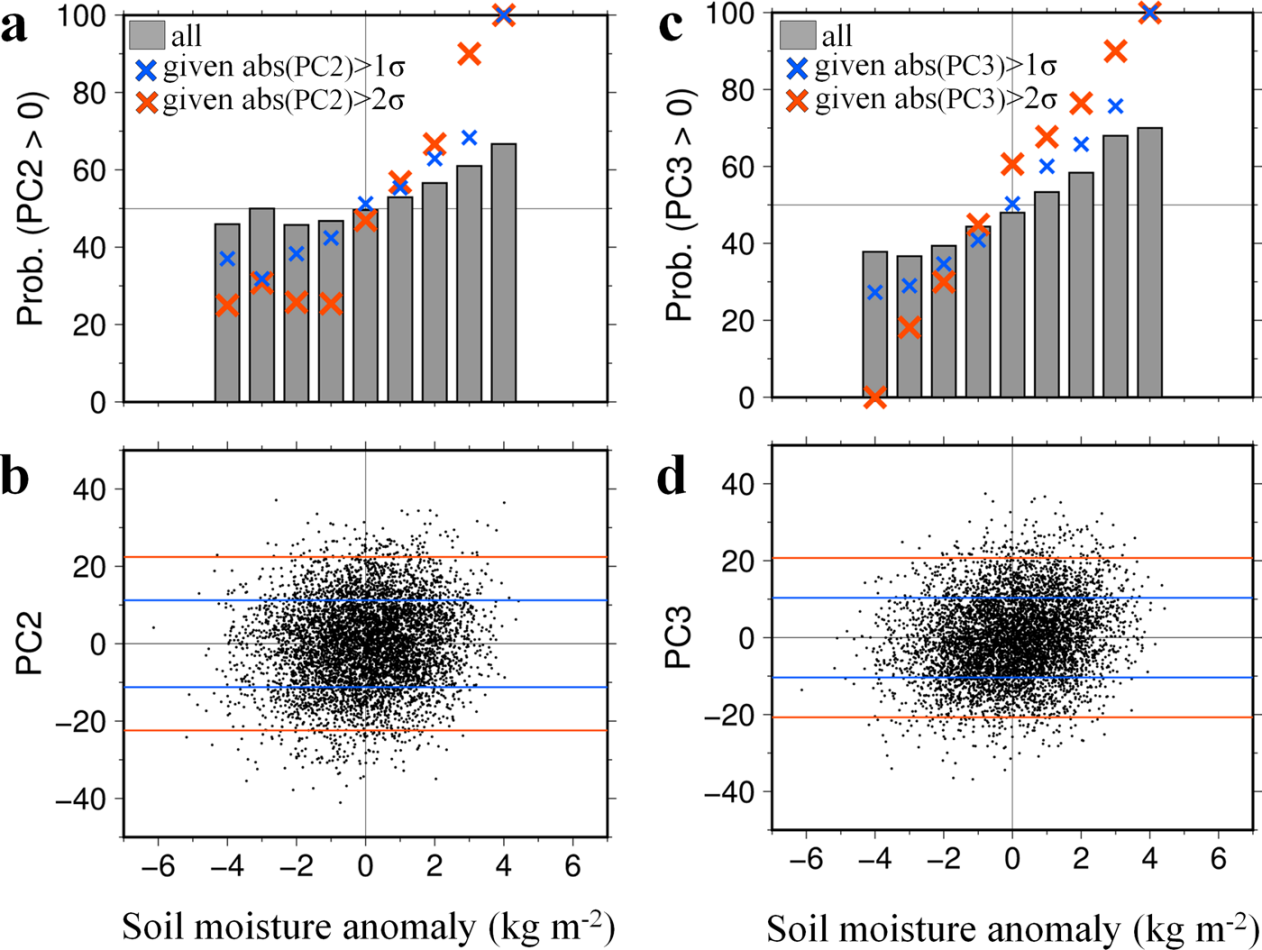
Relationship between summer leading SAT modes and spring soil moisture. (**a**,**c**) Probability that score of (**a**) PC2 and (**c**) PC3 is positive computed for each 1.0 kg m^−2^ interval bin of soil moisture anomalies (bars). Blue and red crosses indicate the probability computed for samples whose deviations from the ensemble average are greater than one and two times the standard deviation, respectively. (**b**,**d**) Scatter plots between the soil moisture anomaly in May averaged over 50°–70°N, 50°–70°E (see Fig. 3), and scores of (**b**) second and (**d**) third PCs for 6,000 samples. The soil moisture anomaly was computed with respect to the ensemble averages. Blue and red lines in (**b**) and (**d**) represent average plus/minus one and two times the standard deviation, respectively.

Soil moisture in May over the Western Siberian Plain (50°–70°N, 50°–70°E) correlates weakly with the PC2 (r = 0.11) and PC3 (r = 0.17) scores (Fig. 6). For both PC2 and PC3, the share of samples with positive scores increases with the soil moisture anomaly. The soil moisture anomaly modulates the diabatic heating of the atmosphere, which helps initiate the quasi-stationary wave^19^. Through the positive feedback of land–atmosphere interactions, the wet soil and low SAT anomalies evolve simultaneously under the low pressure anomaly. The strong dependency of the sign of the PCs on the soil moisture anomaly is obvious for samples with large magnitude scores (exceeding one and two times the standard deviation). This indicates that strong land-to-atmosphere feedback is essential for the maintenance of substantial wave propagation over northern Eurasia. Resonance of PC2 and PC3 with positive sign, which accounts for the recent summer SAT change, is more likely to occur with increasing soil moisture around the Western Siberian Plain in May (Supplementary Fig. 4).

To link the analysis of the huge ensemble experiments with observations, we discuss recent observed changes in snow water. Although the reliability of past changes in snow-related variables over the entire Eurasian continent is controversial, there is observational evidence of recent change in the cryosphere around the Western Siberian Plain. Ground-based measurements have shown that snow depth increased since the 1990s^30^ and that spring snow cover has extended^31^ to around the upper Ob River basin. The number of blocking events and resultant cold events has been increasing over Central Asia^32^. These enhanced snow/cold events appear linked with the reduction of Arctic sea ice since around the late 1990s to 2000s, which modulates the internal dynamics of the atmosphere^33^. It is probable that in recent years quasi-stationary waves have been anchored by positive snow and soil moisture anomalies over central northern Eurasia, which would explain why the summer SAT trend exhibits a tripole pattern. In Fig. 2, time series of PC2 scores exhibits a weak positive shift after the early 1990s, implying that the sea ice reduction modulated the likelihood of a tripole pattern by increasing snowfall events due to enhanced moisture supply from the Arctic Ocean^34^. A recent GCM study demonstrated that snow-fed wet land in spring lowers the summer SAT in the Ob and Volga river basins^35^. Therefore, better prediction of summer SAT anomalies requires sophisticated land surface schemes capable of simulating the delayed discharge of snow water.

The tripole pattern in summer SAT trend is characterised by internal processes involved in the coupled land–atmosphere system. Therefore, multi-GCM averaging or ensemble averaging of model projections might smooth the spatial variability of summer SAT. As shown in Fig. 1c, the wave train-like SAT change in d4PDF disappears due to the ensemble average. It is likely that future change in regional extreme hot events will exceed future projections based on multi-model ensemble averages. Closer monitoring of land surface conditions will be necessary to produce accurate projections of future change in summer SAT. We found that land surface anomalies in spring modulate the likelihood of summer SAT patterns over northern Eurasia. Therefore, our findings can improve the seasonal predictability of abnormal weather in the Eurasian summer.

## Methods

### Data

We analysed the output of a 100-member ensemble AGCM experiment^8^. The output dataset is called the “database for Policy Decision making for Future climate change” (d4PDF), and the variables used in this study are all publicly available (See d4PDF website: http://www.miroc-gcm.jp/~pub/d4PDF/design_en.html). The experiment was performed for the period 1951–2010 using MRI-AGCM3.2 (Meteorological Research Institute AGCM version 3.2)^36^ with TL319 resolution, which approximately corresponds to 60 km resolution. To constrain the model, historical SST, sea ice concentration, and sea ice thickness information was used. The ensemble members were also generated by numerical integration over the same period but with perturbed initial conditions and SST, taking account of analysis error in the observed SST estimates. In addition, concentrations of greenhouse gases, ozone, and aerosols were used for external forcing. Further detailed specifications are available in the dataset description^8^.

In Figs 2 and 3, the Japanese 55-year Reanalysis^37^ (JRA-55) dataset was also used as data independent of d4PDF. Other variables analysed in this study were derived from the d4PDF database unless indicated otherwise. For soil moisture, the surface layer (0–10 cm depth) was used.

### Statistics

The empirical orthogonal function (EOF) analysis was conducted over 22°–90°N, 0°–180°E. For the computation of eigenvectors, a variance-covariance matrix was used. Before the EOF analysis, the d4PDF data was re-gridded from the original 60 km resolution to 240 km. For d4PDF, 6,000 snapshots (60 years, 100 ensemble members) of JJA-averaged SAT were analysed, and the normalised PC scores for the 6,000 snapshots were arranged on the time axis (Fig. 2 and Supplementary Fig. 2). The EOF analysis presented in Supplementary Fig. 2 was based on the detrended SAT in which the deviation of JJA-averaged SAT of each member with respect to the ensemble average was used. In Fig. 4, simulated SAT differences between the 1980s and the 2000s for each ensemble member (n = 100) were analysed. For the EOF analysis using the JRA-55 dataset (Fig. 3), we used the period of 1958–2017.

The reconstructed SAT patterns in Fig. 1b,d were created by the following procedures using d4PDF and JRA-55, respectively. First, regression patterns of JJA SAT against the time series of normalised scores were created for each PC mode (Figs 2a–c and 3a–c). We considered PC1 through PC3 because these modes account for one-third of the total variance in SAT. Second, weighting coefficients for the linear combination were computed for each PC mode. Assuming two matrices corresponding to the spatial distributions of decadal JJA SAT change (Fig. 1a,c) and the regressed SAT pattern (Figs 2a–c and 3a–c), the weighting coefficient was obtained by the inner product of the two matrices. Thus, the weighting coefficient is proportional to the projection of the JJA SAT change pattern onto the eigenvector. The computed weighting factors for the first, second, and third PCs are 2.22, 1.04, and 1.51 for d4PDF, respectively, and 1.80, 1.36, and 0.71 for JRA-55, respectively. These weighting coefficients were chosen to most accurately reproduce the SAT patterns in d4PDF and JRA-55. Finally, the regression patterns from the first to the third modes were overlaid using the weighting coefficients. The same procedures were adopted using the same coefficients as above but for the regression patterns of snow water equivalent and soil moisture (Fig. 5 and Supplementary Fig. 1), and of geopotential height, wind field, and SAT (Supplementary Fig. 3). In Fig. 5 and Supplementary Fig. 3, only PC2 and PC3 were overlaid by setting the coefficient of PC1 to zero, whereas in Supplementary Fig. 1, only PC1 was considered.

## Supplementary information

Supplementary Information
