# Supplementary material for: Intensification of hot Eurasian summers by climate change and land–atmosphere interactions
Source: Sci Rep. 2019 Jul 26;9:10866. doi: 10.1038/s41598-019-47291-5 (PMC6659661; doi:10.1038/s41598-019-47291-5)
Supplement: Supplementary file 1 — Supplementary Information [file 41598_2019_47291_MOESM1_ESM.docx]

**Supplementary Information**

**Intensification of hot Eurasian summers by climate change and land–atmosphere interactions**

Tomonori Sato^1,*^ and Tetsu Nakamura^1^

^1^Hokkaido University, Sapporo, 060-0810, Japan

*Corresponding Author: Tomonori Sato

E-mail: t_sato@ees.hokudai.ac.jp


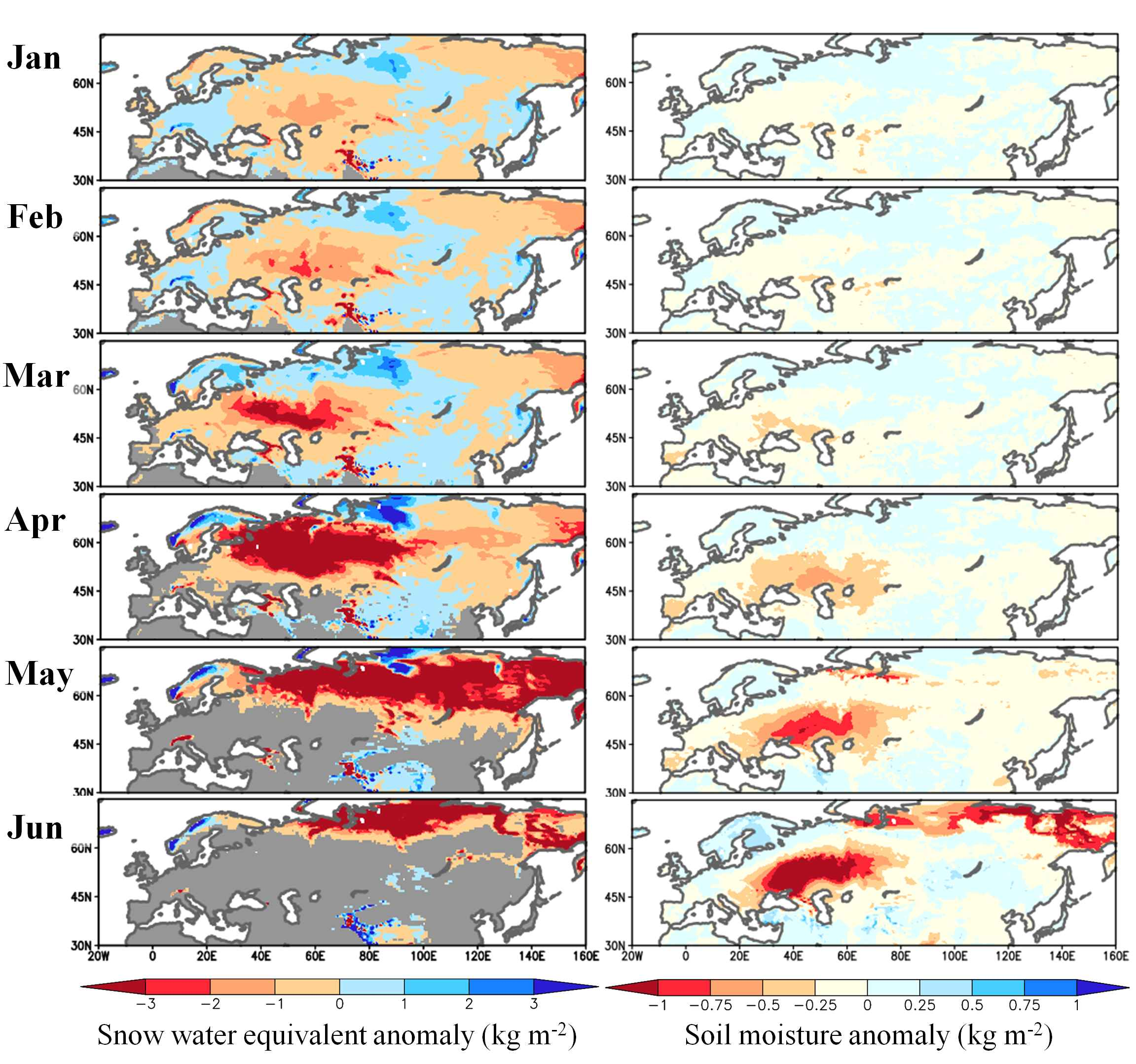


**Supplementary Figure 1: Seasonal variations of snow water equivalent and soil moisture associated with external forcing.**

Regression maps of snow water equivalent **(Left)** and surface soil moisture **(Right)** anomalies against normalised PC1 scores. The grey shading represents the area where climatological snow cover fraction is <0.1. Soil moisture at 0–10 cm depth is presented.


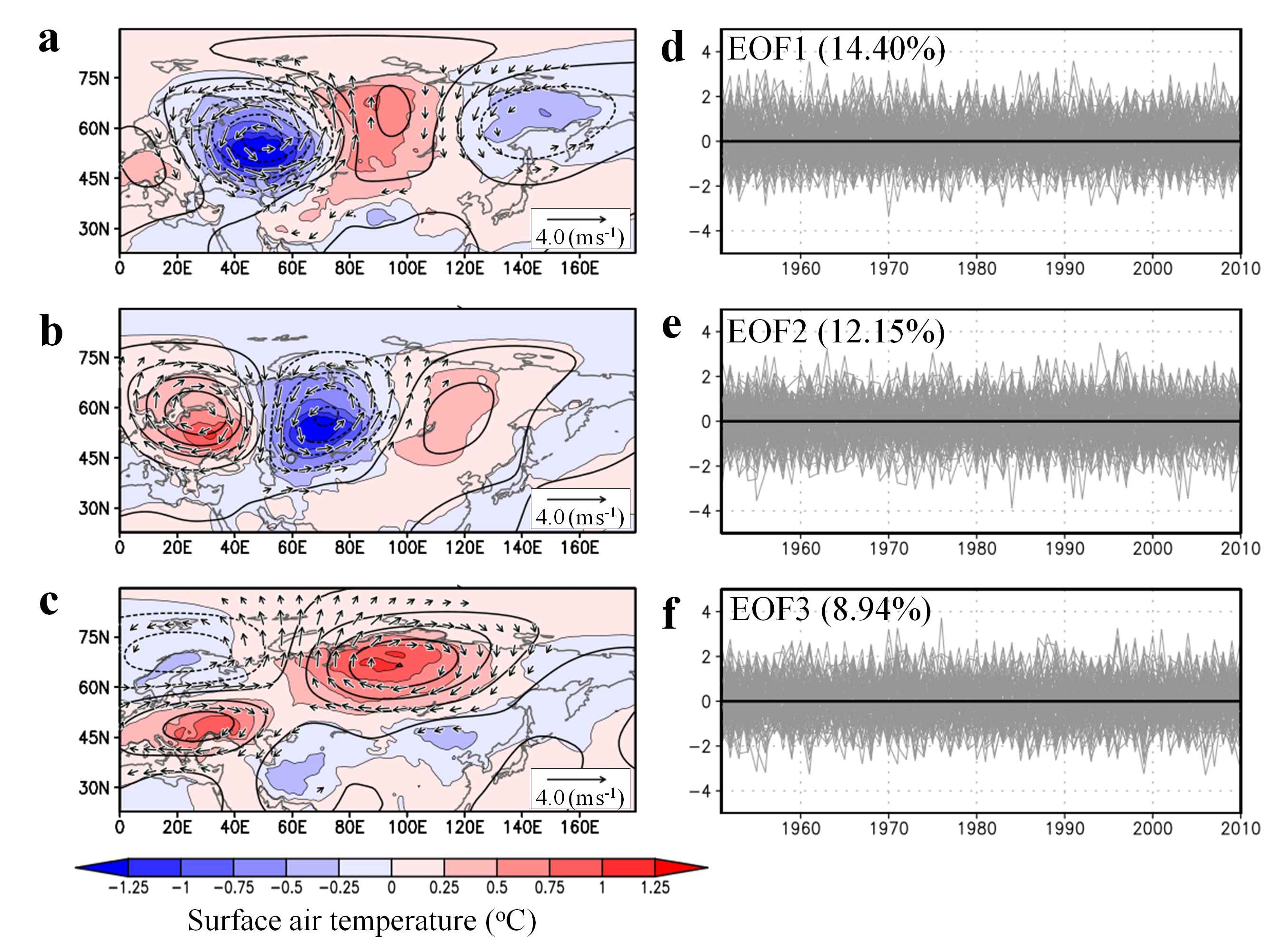


**Supplementary Figure 2: Simulated three leading modes of summer SAT anomalies in d4PDF and their time series associated with internal variability.**

The panels are the same as Fig. 2 but are for the EOF analysis conducted for JJA-averaged SAT anomalies with respect to the ensemble average.


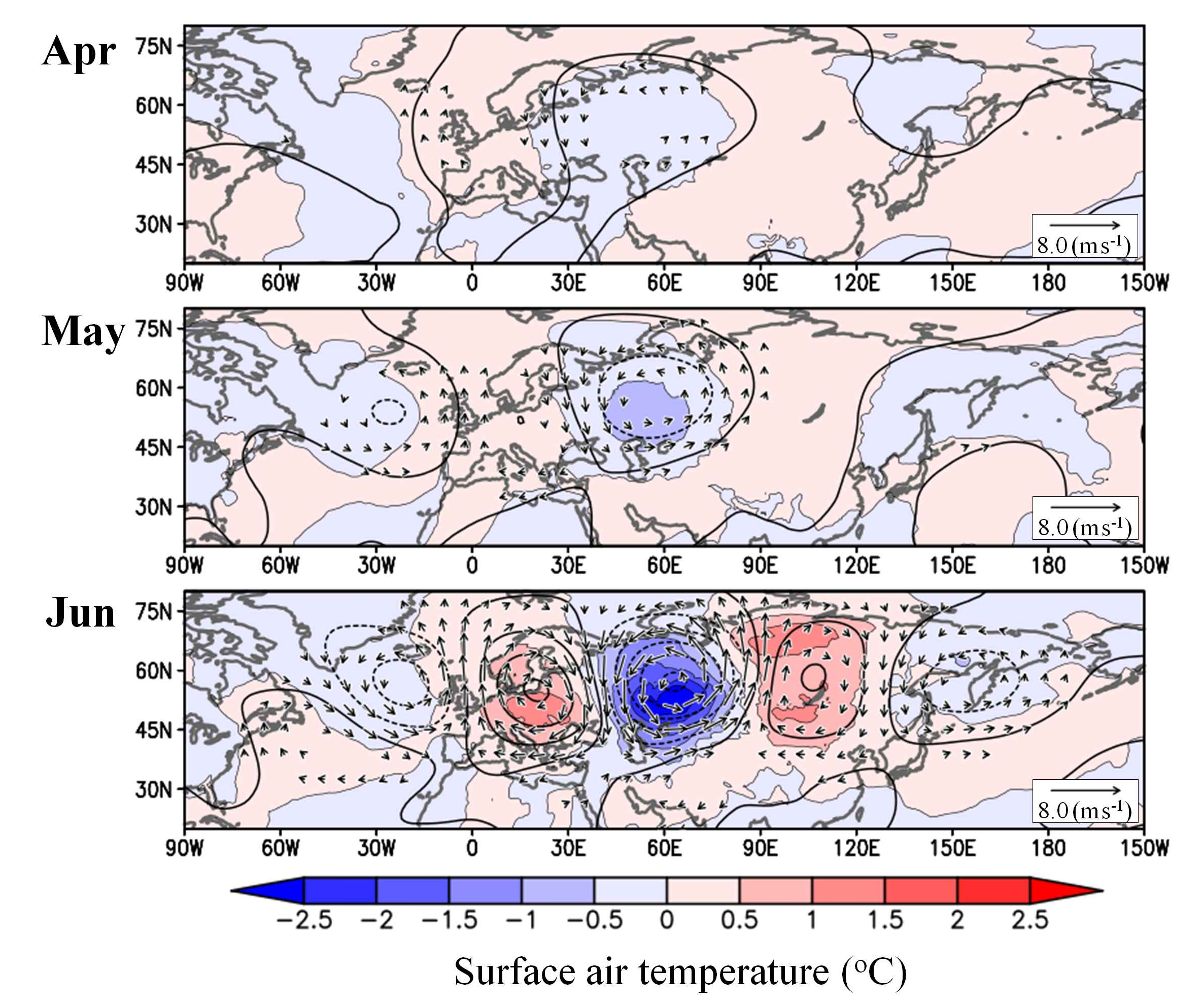


**Supplementary Figure 3: Monthly variations in atmospheric circulation associated with internal forcing.**

The panels are the same as in Fig. 5 but for SAT (shading), 500 hPa geopotential height anomalies (contour: 10 m interval), and wind vectors (wind speeds <0.5 m s^−1^ omitted) derived from d4PDF.


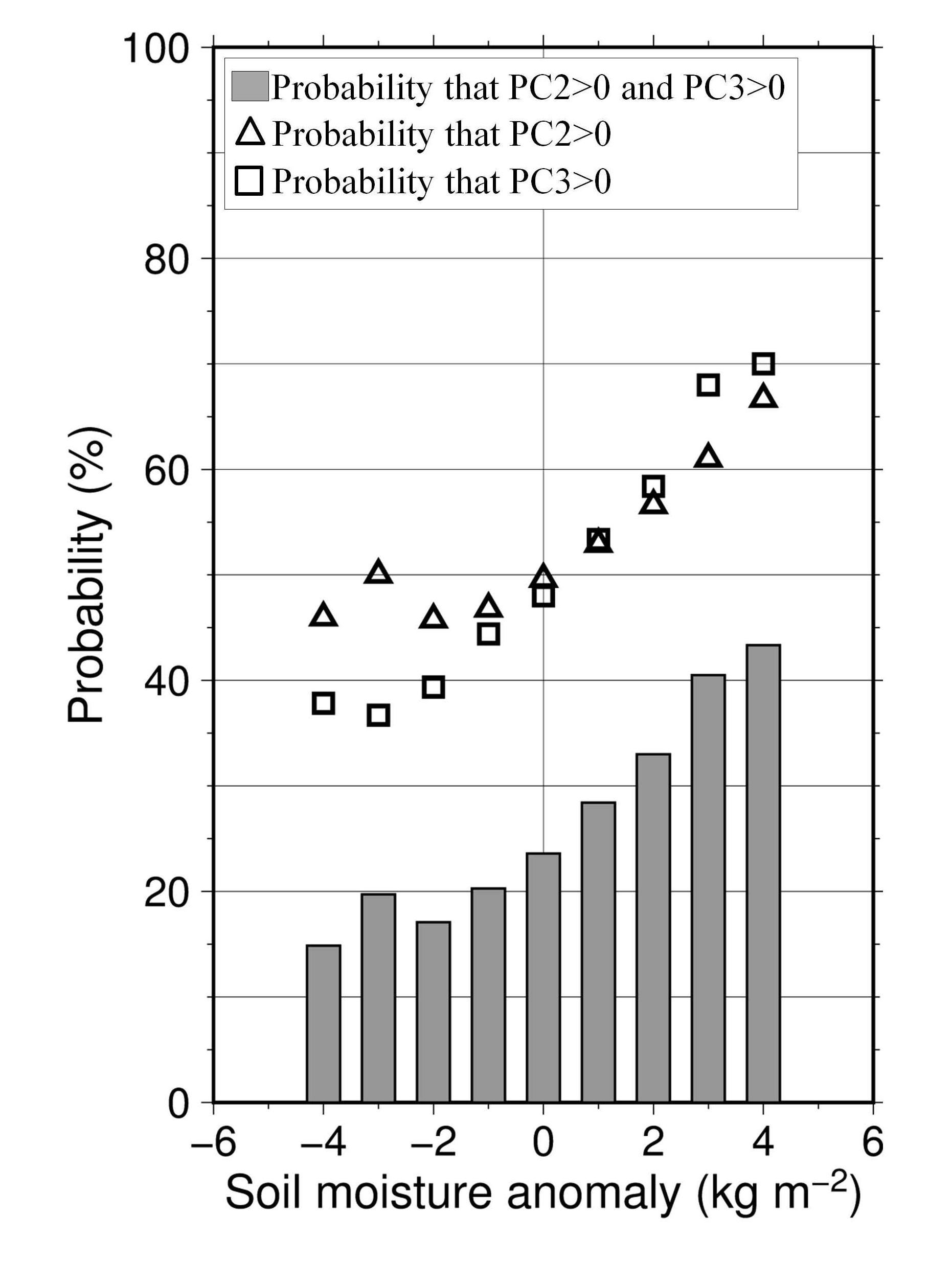


**Supplementary Figure 4: Probability of leading mode resonance affected by spring soil moisture.**

The explanation is the same as in Fig. 6 but for the probability that both PC2 and PC3 are positive (bars) and the probability that PC2 (triangles) and PC3 (squares) are positive.
